# Bone Densitometric Analysis in Egyptian Hemodialysis Patients

**Published:** 2008-06

**Authors:** Ehab I. Mohamed, Eman S. D. Khalil

**Affiliations:** 1 *Department of Medical Biophysics, Medical Research Institute, Alexandria University, Alexandria, Egypt;*; 2 *Department of Nephrology, Medical Research Institute, Alexandria University, Alexandria, Egypt*

**Keywords:** end-stage renal failure, hemodialysis, bone mineral density, dual-enenrgy X-ray absorptiometry, parathyroid hormone, osteocalcin

## Abstract

End-stage renal failure (ESRF) is the ultimate consequence of chronic renal failure, and in such cases dialysis is generally required. Almost all dialysed patients have abnormal bone histology and lower values of glomerular filtration rate have been associated with lower bone mineral density (BMD) at all sites. The objective of the present study was to investigate the effect of hemodialysis (HD) on body-composition (BC), specially segmental and total BMD in Egyptian ESRF patients. Forty ESRF patients (20 male/20 female; age range: 21.00-74.00 yr) undergoing regular HD 3 times/week (duration range: 0.08-20.00 yr) using bicarbonate dialysis and polysulphon membrane were matched with 40 healthy controls for age, height, and sex. Blood samples were obtained and serum levels of calcium (Ca), inorganic phosphate (P), osteocalcin (OC), and parathyroid hormone (PTH) were monitored for all participants. BC was evaluated by dual X-ray absorptiometry. HD patients manifested lower segmental and total BMD values in comparison with age-matched healthy controls (Z-score: -0.17 ± 1.12) due to significantly higher levels of P (4.04 ± 1.33 vs. 3.39 ± 0.51 mg/dl, *p*<0.001), PTH (538.17 ± 363.99 vs. 48.86 ± 19.64 ng/L, *p*<0.0001), and OC (50.39 ± 34.91 vs. 16.32 ± 5.37 μg/L, *p*<0.0001). Pelvis, lumbar spine, and total BMD (g/cm^2^) for HD patients were significantly correlated with HD duration (yr) (R=0.94, 80, and 92, respectively; *p*<0.0001). Thus, BC analysis is of utmost importance for efficiently providing tailored individual mineral supplementation to HD patients.

## INTRODUCTION

End-stage renal failure (ESRF) is the ultimate consequence of chronic renal failure (CRF) and dialysis is generally required until a donor for a renal transplant is found. During progressive renal failure, phosphate retention causes secondary hyperparathyroidism, which leads to hypoplastic bone, characterized by increased bone turnover (1). In the immediate predialysis period, almost all patients have abnormal bone histologies (2), and their lower values of glomerular filtration rate (GFR), and in some studies elevated intact parathyroid hormone (PTH) values, have been associated with lower bone mineral density (BMD) at all sites (3, 4). Additional major bone response patterns include mixed osteodystrophy (hyperparathyroid plus defective mineralization) and low-turnover bone disease (adynamic, aluminum induced, or osteomalacia). Patient age and underlying illness, the degree and period of renal impairment, mode of dialysis, and drug management of hyperparathyroidism, and serum calcium and phosphate influence these patterns of bone histology (5).

Dialysis patients most often are elderly, with numerous comorbid conditions and in poor nutritional health state (6). These patients can also have low body weight and body mass index (BMI), low levels of body fat mass (BFM) and lean body mass (LBM) (7, 8) and have been observed to be at increased risk of osteoporosis (9) and hip fracture (10-13). Hip fractures are a common cause of morbidity and mortality (14). Furthermore, dialysis patients have altered fluid and electrolyte homeostasis (15, 16), which are associated with an increased risk of cardiovascular complications (17, 18), another major cause of morbidity and mortality in renal patients (19, 20). The objective of the present study was to investigate the effect of hemodialysis (HD) on body-composition (BC), specially segmental and total BMD, in Egyptian ESRF patients.

## SUBJECTS AND METHODS

### Subjects

The study population was comprised of 80 subjects, who were divided into two groups. The first [n=40 (20 male/20 female); mean age (± SD) 52.11 ± 12.97 yr, and age range 21.00-74.00 yr] is the study group, which consisted of ESRF patients undergoing regular HD 3 times/week for 4 hours using bicarbonate dialysis to maintain a minimum Kt/V urea index of 1.20 per session. HD patients were followed-up at the Nephrology Department, Medical Research Institute, Alexandria University, Egypt for a mean period of 6.50 ± 5.68 yr and duration range 0.08-20.00 yr. The second (n=40) is the control group, which was matched with the study group for age, height, and sex. All study participants were asked to volunteer to the study and provide signed informed consent prior to their inclusion in the study. The study protocol was in conformity with ethical guidelines of the Medical Research Institute, Alexandria University, Egypt.

HD patients who had an age below 20 years, rheumatic bone disease, history of rickets or osteomalacia before the onset of CRF, history of congenital kidney diseases, history of corticosteroid therapy before the onset of CRF for more than 6 months, history of recurrent kidney transplantation or recent transplantation of less than 6 months, or a positive test for hepatitis B surface antigen or for hepatitis C virus antibodies, were excluded from the study.

All HD patients received bicarbonate dialysis using a low flux polysulphon membrane (F6, Fresenius, Germany), and sessions were conducted under the same standard conditions, with sterile apyrogen delivered water. Blood and dialysate flow-rates were maintained at 250 ml/min and 500 ml/min respectively. The final dialysate concentration was: HCO_3_^-^ 35.0 mM, Na^+^ 140.0 mM, K^+^ 1.5-2.0 mM, Ca^2+^ 1.5 mM. All patients received heparin as a continuous infusion.

### Laboratory Measurements

Complete history was obtained for all participants with emphasis on categorizing bone aches and history of fractures, and complete physical examination with emphasis on bone, joints, and neurological examination. Blood samples were obtained from fasting healthy controls and from HD patients before and after dialysis sessions. Samples were immediately centrifuged and 50 μl of plasma were mixed with 100 μl of 5% metaphosphoric acid. Samples were aliquoted and stored at -80°C until assayed. Blood urea (Ur) and serum creatinine (Cr) were evaluated as detailed elsewhere (21). Serum levels of calcium (Ca) and inorganic phosphate (P) (22), PTH (23, 24), and osteocalcin (OC) (25), were also determined. The Kt/V urea index was calculated according to the formula (26):

(Eq. 1) $$\mathrm{Kt}/V=-\ln\left(R-0.008t\right)+\left(4.0-3.5R\right)\times \mathrm{UF}/W$$

where *R* is the post-dialysis/pre-dialysis blood urea concentration, *t* is the duration of dialysis session in hours, *UF* is the volume of fluid removed during dialysis, and *W* is the post-dialysis weight in kg.

### Body-Composition and Bone Densitometric Measurements

Segmental and total BC [i.e., BFM, LBM, bone mineral content (BMC), and BMD] were measured for all participants using dual-energy X-ray absorptiometry (DXA) (DPX Pro, GE Health Care, USA) at the Department of Medical Biophysics, Medical Research Institute, Alexandria University, Egypt. DXA is the method of choice of BMD for the diagnosis of osteopenia/osteoporosis as recommended by the World Health Organization (WHO) (27).

### Statistical Analysis

Analyses of all data were carried out using the statistical software package StatView 5.0 (SAS Institute Inc., Cary, NC, USA). Descriptive statistics were calculated for the mean ± SD of all relevant variables. Two-tailed *t*-test of significance was used to compare different variables between the HD and control groups. Differences were considered to be significant at *p<0.05*.

## RESULTS AND DISCUSSION

Demographic variables of the study groups and clinical diagnoses of kidney disease of HD patients are presented in Table 1. Diagnosis of the kidney disease was based on clinical and biochemical examinations and echography of all patients, showing that 16 patients (40%) had glomerulonephritis, 8 (20%) had vascular nephropathy, 12 (30%) had diabetes type II, 2 (5%) had polycystic kidney disease, while 2 patients (5%) had shown other causes.

**Table 1:** Demographic and clinical diagnosis of kidney disease data for hemodialysis patients (*n=40*) and healthy controls (*n=40*)

	Hemodialysis	Control

**Age** *(year)*	52.11 ± 12.97	51.64 ± 12.95
**Sex** *(Male/Female)*	20/20	20/20
**Height** *(m)*	1.57 ± 0.09	1.64 ± 0.10
**Weight** *(kg)*	66.36 ± 15.44^a^	84.05 ± 17.51
**Body Mass Index** *(BMI, kg/m^2^)*	26.79 ± 5.89^a^	31.30 ± 6.15
**Kidney Disease**		
*** Glomerulonephritis***	16 (40%)	—
*** Vascular Nephropathy***	8 (20%)	—
*** Diabetes Type II***	12 (30%)	—
*** Polycystic Kidney Disease***	2 (5%)	—
*** Others***	2 (5%)	—

Values are Mean ± SD.

a *P*<0.0001 as compared to the Control group.

**Figure 1:**
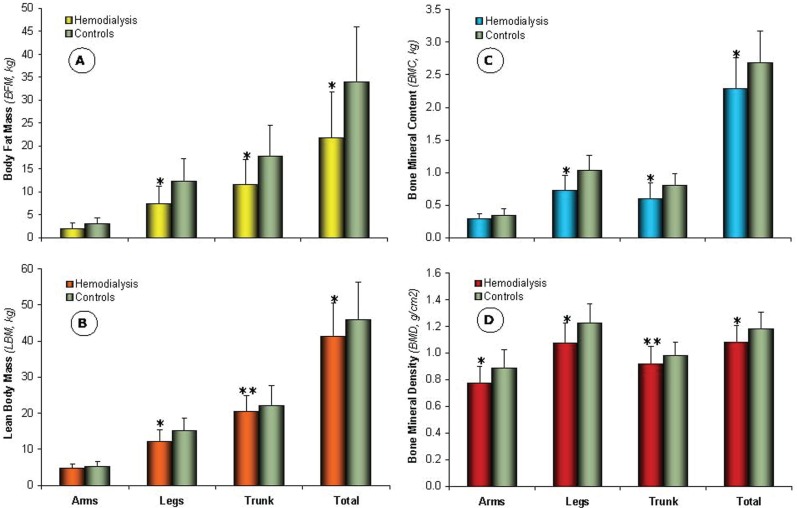
Graphical plot of segmental (i.e., arms, legs, and trunk) and total body-composition analysis for hemodialysis patients (*n=40*) in comparison with healthy controls (*n*=40). **A,** Body Fat Mass (BFM, *kg*); **B,** Lean Body Mass (LBM, *kg*); **C,** Bone Mineral Content (BMC, *kg*); **D,** Bone Mineral Density (BMD, *g/cm^2^*). Data are presented as mean ± SD. ^*^P<0.001 and ^**^P<0.05 as compared to healthy controls.

Both weight and BMI were significantly lower for HD patients as compared to healthy controls (66.36 ± 15.44 and 26.79 ± 5.89 vs. 84.05 ± 17.51 kg and 31.30 ± 6.15 kg/m^2^, respectively, *p*<0.001), as a direct consequence of long durations of dialysis reaching 20 yr in some patients. The BC data for study groups are shown in Figure 1. The segmental (i.e., arms, legs, and trunk) and total BFM for HD patients (Figure 1A) were significantly lower (*p*<0.001), except for arms BFM, (2.04 ± 1.13, 7.42 ± 3.82, 11.63 ± 5.53, and 21.79 ± 10.05 kg, respectively) as compared to healthy controls (3.06 ± 1.30, 12.30 ± 4.85, 17.74 ± 6.71, and 33.97 ± 12.04 kg, respectively). The same trend of difference between the two groups was also observed for segmental and total LBM, BMC, and BMD (Figure 1B, 1C, and 1D, respectively). These data are in line with previous findings showing that dialysis patients are in poor nutritional health state evidenced by low body weight, BMI, BFM, and LBM (6-8), thus being at an increased risk of osteoporosis and hip fracture (9-13).

Serum levels of biochemical markers of renal function of both studied groups are shown in Table 2. Pre-dialysis blood Ur and Cr for HD patients were significantly higher than those for healthy controls (123.51 ± 40.92 and 10.91 ± 2.61 vs. 32.07 ± 8.12 and 0.88 ± 0.17 mg/dl, respectively, *p*<0.0001). While serum Ca levels were comparable in both groups, inorganic P levels for HD patients were significantly higher than those for healthy controls (4.04 ± 1.33 vs. 3.39 ± 0.51 mg/dl, *p*<0.05), which resulted in a significantly higher (*p*<0.05) Ca × P product. PTH and OC levels were also significantly higher for HD patients as compared to healthy controls (538.17 ± 363.99 and 50.39 ± 34.91 vs. 48.86 ± 19.64 ng/L and 16.32 ± 5.37 μg/L, respectively, *p*<0.0001). The evident variability of PTH levels for HD patients, which ranged from 93.90 to 1594.00 ng/L, may derive from the degree of CRF, the duration of dialysis, and the type of dialysis membrane, which are mainly affected by the socioeconomic level of Egyptian patients. Due to poverty, Egyptian CRF patients tend to be referred for dialysis very late, usually at Cr clearance levels below 10 ml/min, and they typically cannot afford expensive phosphate binders. Phosphate retention during progressive renal failure has been shown to cause secondary hyperparathyroidism (i.e., increased levels of PTH), leading to hypoplastic bones, characterized by increased bone turnover (1). Moreover, altered fluid and electrolyte homeostasis in dialysis patients has been shown to be associated with an increased risk of cardiovascular complications, one of the major causes of morbidity and mortality in renal patients (15-18).

**Table 2:** Blood biochemical data for hemodialysis patients (*n=40*) and healthy controls (*n=40*)

	Hemodialysis	Control

**Hemodialysis Duration** *(year)*	6.50 ± 5.68	—
**Pre-Dialysis Urea** *(Pre-Ur, mg/dl)*	123.51 ± 40.92^a^	32.07 ± 8.12
**Kt/V**	1.21 ± 0.08	—
**Creatinine** *(Cr, mg/dl)*	10.91 ± 2.61^a^	0.88 ± 0.17
**Calcium** *(Ca, mg/dl)*	8.72 ± 1.03	8.99 ± 0.53
**Inorganic Phosphate** *(P, mg/dl)*	4.04 ± 1.33^b^	3.39 ± 0.51
**Ca × P** *(mg^2^/dl^2^)*	34.52 ± 9.62^b^	30.39 ± 4.47
**Parathormone** *(PTH, ng/L)*	538.17 ± 363.99^a^	48.86 ± 19.64
**Osteocalcin** *(OC, μg/L)*	50.39 ± 34.91^a^	16.32 ± 5.37

Values are *Mean ± SD.*

a *P*<0.0001;

b *P*<0.05 as compared to the Control group.

In depth analysis of BMD at all sites showed that arms, legs, pelvis, and total BMD (Table 3) were significantly lower (*p*<0.001) for HD patients as compared to healthy controls. Overall T- and Z-Scores for HD patients were significantly lower than those for healthy controls (-0.44 ± 1.26 and -0.17 ± 1.12 vs. 0.57 ± 1.21 and 0.28 ± 1.01, respectively, *p*<0.001). Moreover, pelvis, lumbar spine; representing two of the sites more susceptible to fracture risk; and total BMD for HD patients were significantly correlated with HD duration (R=0.94, 80, and 92, respectively, *p*<0.0001). Earlier studies have shown that lower values of GFR associated with elevated intact PTH values were associated with lower BMD at all sites (3, 4), as demonstrated by abnormal bone histology for patients in the immediate pre-dialysis period (2). These patterns of bone histology were influenced by patient age and underlying illness, the degree and period of renal impairment, mode of dialysis, and drug management of hyperparathyroidism, and serum Ca and inorganic P (5).

**Table 3:** Segmental and total Bone Mineral Density (BMD, g/cm^2^) data together with T- and Z-Scores for hemodialysis patients (*n=40*) and healthy controls (*n=40*)

	Hemodialysis	Control

**Bone Mineral Density** (*g/cm^2^*)		
***Head***	2.23 ± 0.37	2.26 ± 0.31
***Arms***	0.77 ± 0.13^a^	0.89 ± 0.13
***Legs***	1.08 ± 0.15^a^	1.22 ± 0.14
***Trunk***	0.92 ± 0.13	0.98 ± 0.10
***Ribs***	0.71 ± 0.10	0.77 ± 0.09
***Pelvis***	1.05 ± 0.18^b^	1.16 ± 0.14
***Lumbar Spine***	1.15 ± 0.24	1.16 ± 0.16
***Total***	1.08 ± 0.13^a^	1.18 ± 0.12
**T-Score**	- 0.44 ± 1.26^a^	0.57 ± 1.21
**Z-Score**	- 0.17 ± 1.12^a^	0.28 ± 1.01

Values are Mean ± SD.

a *P*<0.001;

b *P* < 0.05 as compared to the Control group.

In conclusion long term HD is associated with alteration of fluid and electrolyte homeostasis in ESRF patients. That is, observed P retention and elevated Ca × P product, which causes secondary hyperparathyroidism leading to significant lowering of segmental and total BMD. Thus, BC analysis is of utmost importance for efficiently providing tailored individual mineral supplementation during dialysis sessions to HD patients.
